# Applying Deep Learning to Quantify Drivers of Long‐Term Ecological Change in a Swedish Marine Protected Area

**DOI:** 10.1002/ece3.72091

**Published:** 2025-09-02

**Authors:** Christian L. Nilsson, Søren Faurby, Emil Burman, Jurie Germishuys, Matthias Obst

**Affiliations:** ^1^ Department of Marine Sciences University of Gothenburg Gothenburg Sweden; ^2^ Gothenburg Global Biodiversity Centre Gothenburg Sweden; ^3^ Department of Biological and Environmental Sciences University of Gothenburg Gothenburg Sweden; ^4^ Combine AB Gothenburg Sweden

**Keywords:** deep learning, hard‐substrate communities, long‐term ecological trends, marine protected areas (MPAs), object detection models, video‐based biodiversity monitoring

## Abstract

In this study, we trained an object‐detection model to classify 17 benthic invertebrate taxa in archived footage of a study site on the northern west coast of Sweden (a wall section of the Koster Fjord) within the Swedish marine protected area Kosterhavet National Park. The model displayed a mean average precision score of 0.738 and was applied to footage from 1997 to 2023, generating a dataset of 72,369 occurrence records. The dataset was used to quantify depth distributions and abundance trends of both individual taxa and functional groups over time. Depth distributions for 15 of 17 taxa occurred at depths ≥ 45 m. Distributions of 11 taxa aligned with empirical observations, and for the remaining six taxa, we propose expanded depth distributions in the area. Abundances over time significantly increased for eight taxa and decreased for five taxa, while the overall community structure throughout the study period shifted toward smaller, more heat‐tolerant suspension feeders. We found that temperature preference and size were significant drivers of the observed abundance trends in individual taxa. Community structure was altered by the loss of large, heat‐sensitive taxa to greater depths due to increased temperatures. We also observed a strong trend of increasing abundances in the remaining community, including six trawling‐sensitive taxa, highlighting the effectiveness of the park's protective measures. To protect key cold‐water species, we suggest that current fishery regulations of the national park should be expanded to deeper (colder) waters and that new marine protected areas should also be established in deep waters. Our study demonstrates the application potential of video surveillance combined with deep‐learning technology, and we recommend the implementation of standardized video monitoring in marine ecosystem management.

## Graphical abstract

We applied an object‐detection model to archived videos from 1997‐2023 of a submarine rock wall in a Swedish marine protected area. We modeled depth distributions and abundance trends of 17 invertebrate taxa. Most taxa resided at deeper wall sections and abundance trends were generally positive, but heat sensitivity was associated with population decline. This highlights a partially successful management strategy of the protected area that should be adapted to preserve key cold‐water species.

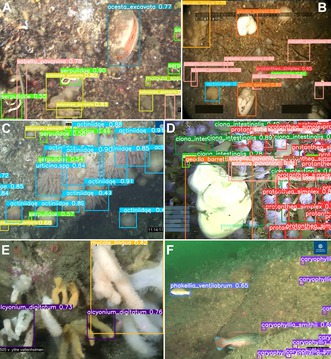

## 1 Introduction

Societal development and human population growth of the past centuries have caused marine environments to face unprecedented anthropogenically induced pressures (Feist and Levin 2016; Moranta et al. 2022). Industrial fisheries overexploit fishing grounds for short‐term economic gains, leading to widespread habitat destruction, oftentimes causing ecosystem collapse (Kemp et al. 2023; Turner et al. 1999). In parallel, climate change is shifting distribution ranges of marine species poleward and to greater depths to retain their climatic niche, restructuring food webs globally (Burrows et al. 2019; Pinsky et al. 2020). Climate‐change driven range shifts and anthropogenic stressors are also reflected in biodiversity at a local scale (Elahi et al. 2015). Therefore, the protection of marine environments at local scales is an important measure to maintain biodiversity and ecosystem functioning against the ongoing trend of biodiversity loss worldwide (Topor et al. 2019).

Through international collaboration, large initiatives have started to protect and restore biodiversity. However, they are often hindered by an undersupply of biodiversity data at a local scale (Bowler et al. 2024; CBD 2010; European Parliament 2024). This increases the risks of misguided management practices and failures of expensive restoration efforts (Bowler et al. 2024; Ditria et al. 2022). Ideally, the foundation of local biodiversity data should also cover large temporal scales to discern changes in biodiversity caused by climatic shifts and/or anthropogenic activities from background oscillations (Sukhotin and Berger 2013). To this end, the concept of essential biodiversity variables (often referred to as EBVs) has been established (Pereira et al. 2013) with the goal to provide critical variables required to study, report, and manage biodiversity change. However, for most marine species and ecosystems, essential biodiversity variables cannot be produced due to the lack of cost‐efficient methods for monitoring and data collection (Kissling et al. 2018; Painting et al. 2020). Detailed and efficient monitoring is hence key to managing biodiversity through protection of key environments and restorative efforts (Ditria et al. 2022; González‐Rivero et al. 2020).

Significant knowledge gaps can be found regarding benthic invertebrate communities, which, despite making up 92% of marine species, are often underrepresented in IUCN reports and national conservation efforts. Furthermore, marine invertebrates play essential roles in ecosystem functioning, such as nutrient cycling, habitat engineering, and water quality improvement. Thus, marine invertebrates help maintain a continuation of ecosystem services that have ultimately benefited humanity (Chen 2021). For a few economically important (primarily vertebrate) species, fisheries data have provided detailed accounts of historical abundances and distributions, allowing for more well‐informed management of their populations (Barbeaux et al. 2020; Caputi et al. 2016; Hare and Able 2007; Perry et al. 2005). However, for the less commercially exploited majority of marine invertebrates, there is an urgent need for further assessment to pinpoint site‐ and species‐specific conservation (Mather 2013; Snelgrove 2016).

Marine invertebrates themselves hold high value for environmental monitoring purposes because shifts in their composition often reflect wider patterns of human impact or natural disturbances (Steyaert et al. 2020). Using trawling as an example, sessile suspension‐feeding organisms can be negatively impacted by resuspended sediments obstructing their feeding and potentially smothering them (González‐Irusta et al. 2018). Large organisms also take longer to recover from physical disturbances and sediment smothering due to their slower growth rate (Hinz et al. 2021) but are also selected against under warming conditions (Peck et al. 2009). However, applying these indicators to hard‐substrate communities can be difficult since well‐studied organisms with clear traits data generally stem from easily investigated habitats, for example, soft substrates in coastal environments (Chen 2021; Robert et al. 2017). A research bias toward certain habitats and taxa may therefore cause many ongoing ecological trends to be left unnoticed, with baselines being shifted to a state of less biodiversity.

Rock walls are an understudied habitat type, highly important to benthic invertebrates (Robert et al. 2015, 2017; Thomasson and Tunberg 2005). The exposed hard substrate in these habitats provides anchoring points for sessile fauna, which in turn shelter mobile fauna. High water flow increases the flux of food and larvae to the rock wall, while steep topography prevents sediment accumulation and provides shade, reducing spatial competition with algae (Gasbarro et al. 2018; Miller and Etter 2011). Along the wall's depth gradient, environmental conditions (e.g., temperature, salinity, pressure, light, and food availability) change, giving rise to high vertical heterogeneity in community structure, enabling the formation of diverse communities over small horizontal scales (Kaiser et al. 2020; Stotz et al. 2016). The steep topography of rock walls can offer natural protection from anthropogenic mechanical disturbances, such as trawling. However, it also complicates environmental surveying (Robert et al. 2017). Therefore, scientific studies of rock walls have primarily been conducted at depths accessible by SCUBA equipment (Altieri and Witman 2014; Cárdenas and Montiel 2015; Thomasson and Tunberg 2005), while studies investigating deeper rock wall communities have been few and primarily focused on qualitative descriptions (Gasbarro et al. 2018; Stotz et al. 2016).

Recent advances in camera and Remotely Operated Vehicle (ROV) technology have opened deep rock wall habitats for detailed visual surveying through a non‐invasive approach (Robert et al. 2017). This has caused a significant increase in data quantity, with several recent ecological investigations presenting large imagery‐based biodiversity assessments from historically inaccessible habitats (Pearman et al. 2020; Rabone et al. 2023; Robert et al. 2015, 2017; Simon‐Lledó et al. 2020; Yamakita et al. 2018). Extracting data from archived footage can also expand temporal scales of local biodiversity assessments, facilitating the tracking of ongoing biodiversity trends (Aguzzi et al. 2020; Bonofiglio et al. 2022). While it is possible to generate vast quantities of organism occurrences through video monitoring, the method faces a strict bottleneck from arduous manual processing (Cuvelier et al. 2024; Lürig et al. 2021; Villon et al. 2022).

Deep‐learning algorithms have enabled the automation of visual biodiversity assessments through computer vision (Lürig et al. 2021; Villon et al. 2022), causing a recent revolution in video‐based monitoring. Several successful ecological applications of deep learning and computer vision have been documented. For instance, González‐Rivero et al. (2020) trained VGG‐D 16, a Convolutional Neural Network (CNN) architecture to estimate benthic cover of five functional groups in coral reefs and found 97% agreement between machine and expert estimations. Villon et al. (2018) trained the CNN architecture GoogLeNet to distinguish between 20 coral reef fish species with an accuracy of 78%. Cuvelier et al. (2024) utilized the architecture Faster R‐CNN to distinguish between 57 deep‐sea taxa with an accuracy of > 90%. Liang and Song (2023) trained a lightweight object‐detection algorithm from the YOLOv5 architecture to detect four classes of small marine invertebrates in real time, with a mean Average Precision (mAP; Equation 5) of 85.1%. Automating these surveys have led to savings in both labor and time, with the case of González‐Rivero et al. (2020) leading to an estimated 99% cost reduction and 200 times accelerated processing time. Thus, computer vision and deep learning allow marine ecologists to embrace big data, establishing a new toolbox that can help quantify ecological trends and link to potential stressors, facilitating sustainable management of marine ecosystems.

In this study, we aim to apply a deep‐learning algorithm to process archived ROV footage for quantitative investigations of the community structure and ecological trends at a rock‐wall section in a Swedish Marine Protected Area (MPA). This was done by: (1) Training an object‐detection model to detect prominent invertebrate fauna at the study site and evaluate its performance. (2) Estimating upper and lower bounds of depth distributions for investigated taxa at the study site, based on the distribution of their abundance. (3) Quantifying temporal ecological trends and their drivers by modeling abundance trends of different taxonomic/functional groups and correlating trends to traits of temperature and fishing sensitivity. (4) Based on our findings, provide recommendations to policymakers on efficient and effective management of the MPA and its associated communities.

## 2 Methods

### 2.1 Study Site and Video Material

**FIGURE 1:**
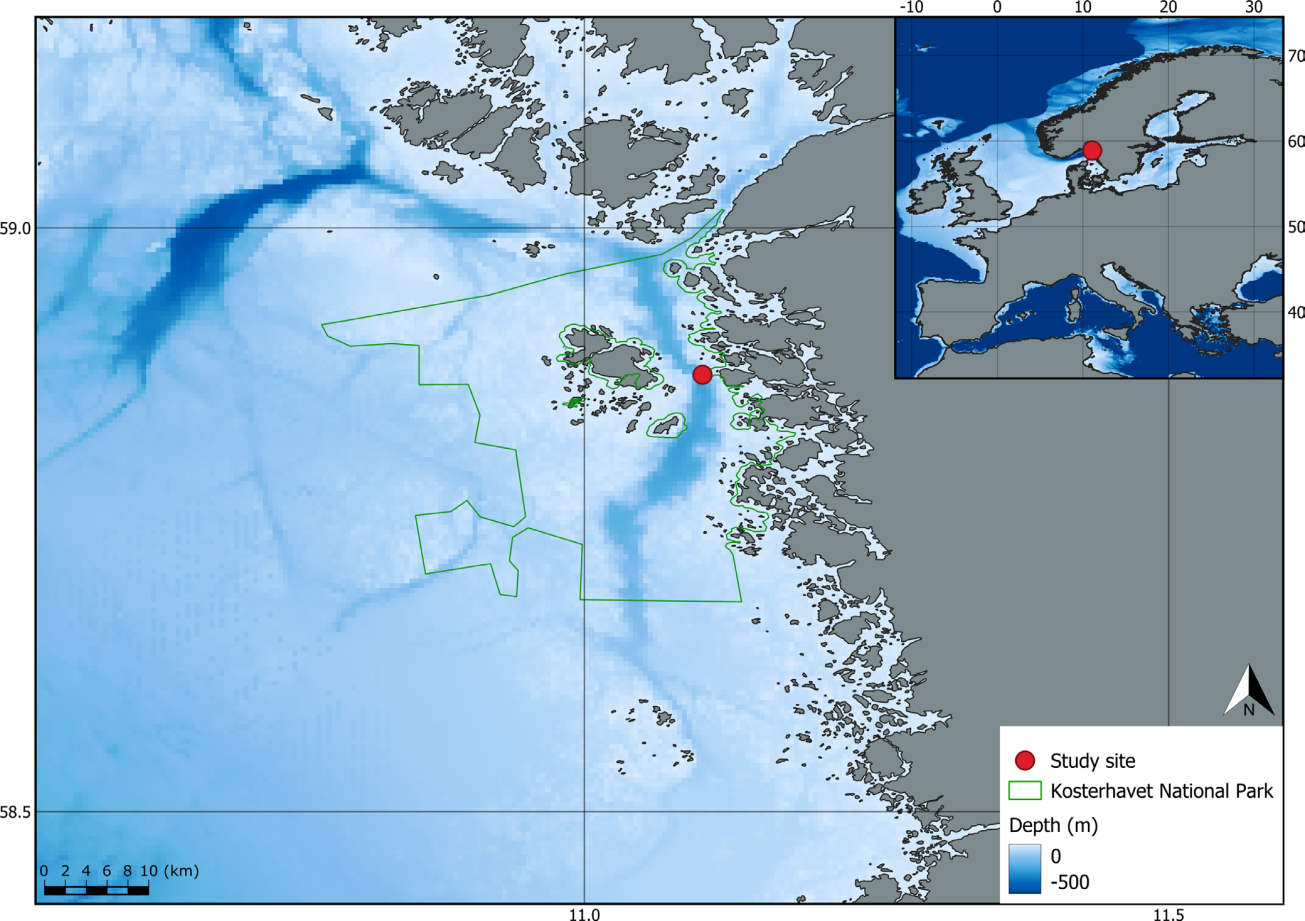
Map showing geographic location of the study site and bathymetry of the surrounding area, with depths ≥ 500 m given the same color. Bathymetry data from GEBCO Compilation Group (2024).

**FIGURE 2:**
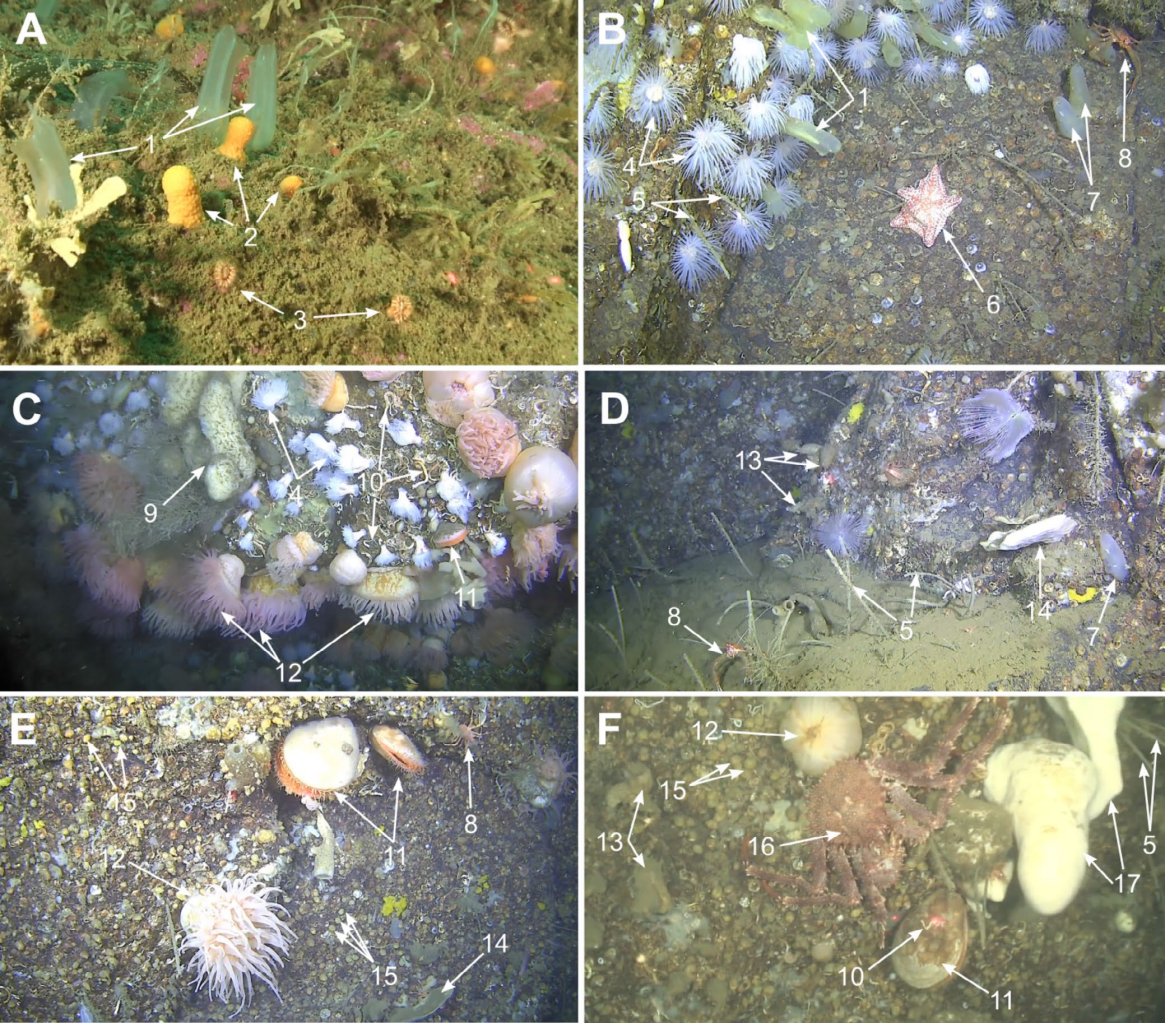
Habitats from the study site and associated fauna which the model was trained to detect. (A) Shallow slopes. (B) Wall/slope from shallow‐intermediate section. (C) Wall and overhang from intermediate‐deep section. (D) Wall and ledge from deep section. (E) Wall and overhang from bottom section. (F) Wall from bottom section. 1, *Ciona intestinalis* ; 2, *Alcyonium digitatum* ; 3, *Caryophyllia smithii* ; 4, *Protanthea simplex* ; 5, *Sabella pavonina* ; 6, *Porania pulvillus* ; 7, *Ascidia* spp.; 8, *Munida* spp.; 9, *Mycale lingua* ; 10, Serpulidae; 11, *Acesta excavata* ; 12, Actiniidae; 13, *Polycarpa pomaria* ; 14, *Phakellia ventilabrum* ; 15, *Molgula* spp.; 16, *Lithodes maja* ; 17, *Geodia barretti*. Photos: University of Gothenburg, Tjärnö Marine Laboratory.

The study was conducted in the marine protected area (MPA), Kosterhavet National Park located in the northernmost part of Sweden's west coast, covering nearly 400 km^2^ (Figure 1). Tullroth et al. (2019) described the national park as being heavily influenced by the 247 m deep Koster Fjord, and Polovodova Asteman et al. (2021) documented the Koster Fjord's connection to the Norwegian trench as a supply of cold, saline, well‐oxygenated water carrying nutrients to the area. This water supply also acts as a source for larvae of rare deep‐dwelling species (Tullroth et al. 2019). Both Tullroth et al. (2019) and Gonzalez‐Mirelis et al. (2009) document rich and diverse invertebrate communities on the fjord's walls, with several occurrences of rare species (Figure 2). The MPA received Natura 2000 Site of Community Importance (SCI) status in January 2005 and Special Area of Conservation (SAC) status in March 2011 as a Natura 2000 protected area. In 2009, it was also protected as a national park, making the Koster Fjord part of Sweden's first marine national park. Seafloor trawling for shrimp is still permitted in the MPA; however, it is heavily regulated. All trawling is banned at depths < 60 m, and six trawling‐free zones at > 60 m depth were established in 2009, with five additional zones being established in 2015 (one directly at the study site) (Tullroth et al. 2019).

**FIGURE A1:**
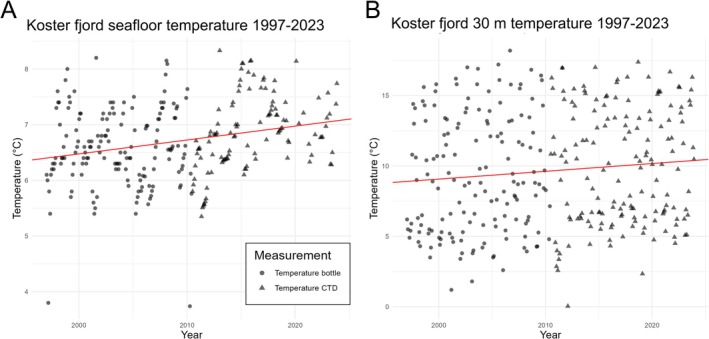
Linear regressions on temperatures measured by the Swedish Meteorological and Hydrological Institute at station “KOSTERFJORDEN NR16” against time (days) from years investigated in this study. (A) Seafloor temperature (estimate = 6.84 × 10^−5^; std. error = 1.55 × 10^−5^; *r* = 0.260; *p* < 0.001). (B) Temperature at 30 m depth (estimate = 1.51 10^−5^e‐4; std. erro*r* = 8.14 × 10^−5^; *r* = 0.105; *p* = 0.0645). Data collected from SHARKWEB (https://shark.smhi.se/hamta‐data/) (Accessed 23‐01‐2025).

Trawling likely reduces the area's biodiversity since a local report comparing benthic biodiversity in the MPA reported a higher taxon diversity at sites without evidence of trawling than at comparable sites with trawling marks (Jonsson 2014). Furthermore, trawling likely has a negative impact on fauna sensitive to sedimentation, even in trawling‐free zones (Linders et al. 2014). Another local report by Linders et al. (2014), investigating the impact of trawling on turbidity in the Kosterhavet National Park, documented elevated turbidity (primarily at > 60 m depth, where trawling is permitted) in one of the MPA's trawling‐free zones on days when trawling was carried out. The area has also been subject to environmental changes, with recent trends including significant increases in temperature at the deepest point of the fjord (Figure A1A in Appendix A), near‐significant increases in temperature at shallow depths (Figure A1B in Appendix A), and desalination of surface waters (Polovodova Asteman et al. 2021). Thus, the Kosterhavet National Park has, despite its protective measures, experienced several direct and indirect anthropogenic pressures over the past three decades.

The Koster Fjord has received extensive Remotely Operated Vehicle (ROV) coverage over the past 30 years. However, this footage mainly consists of non‐standardized, archived material collected for various purposes. Gonzalez‐Mirelis et al. (2009) describe the footage as “opportunistic” due to it not being gathered to map community structure nor being collected from reproducible transects. This caused several challenges when extracting community structure data from these videos. Problems included: variable distance to substrate, variable ROV flying speed, stopping at “interesting” fauna, winding surveying tracks, and angled filming causing mismatches between ROV depth and seafloor depth. Lack of a video overlay containing depth information also caused several videos to be completely discarded from this study. However, spatial inconsistencies in the footage are effectively removed by our approach focusing on a single site (Figure 1). Furthermore, the aforementioned artifacts are outweighed by a wealth of information available in the investigated footage, which has already proven useful for a semiquantitative manual assessment of the MPA's communities (Gonzalez‐Mirelis et al. 2009).

**FIGURE A2:**
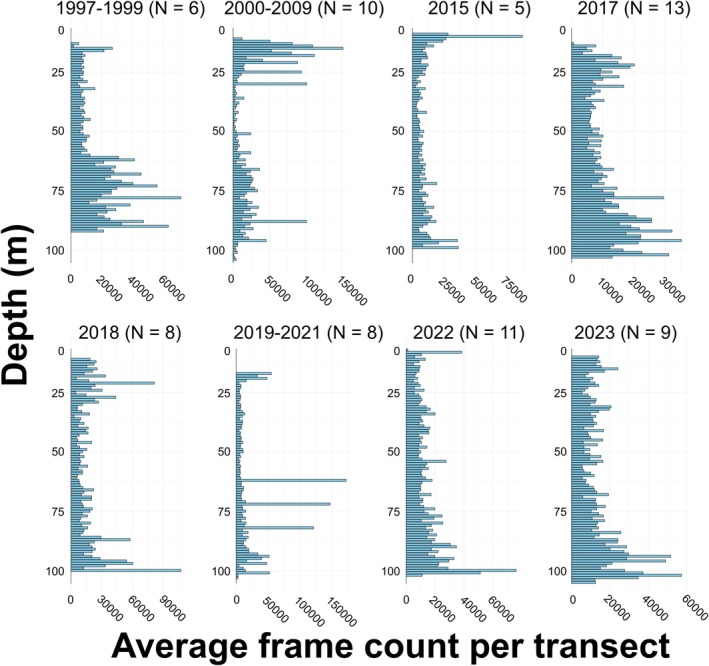
Histograms of video frame counts per depth unit. Averaged by transects per time‐period for visualization purposes.

The habitats investigated in this study were hard substrate habitats of the slope (7 m–~45 m) and wall (~45 m–105 m) west of Yttre Vattenholmen (Figures 1 and 2). A total of 70 transects (defined as consecutive filming of the study site until departure) were chosen from the years 1997–2023 with varying coverage of the study site's 7–105 m depth gradient (Figure A2 in Appendix A). Transect frequency averaged at 1–2 transects per year between 1997 and 2009 and 4–5 per year between 2015 and 2023 (Table A1). Footage was curated from the archives of Tjärnö Marine Laboratory and uploaded to SUBSIM^1^, the Swedish platform for subsea image analysis (Anton et al. 2021) for image annotation, model training, and analysis. Here, a total of 4,487,105 frames were analyzed, with each frame being assigned a total number (including 0) of modeled detections for each of the 17 investigated taxa (Figure 2).

### 2.2 Model Training and Evaluation

**FIGURE 3:**
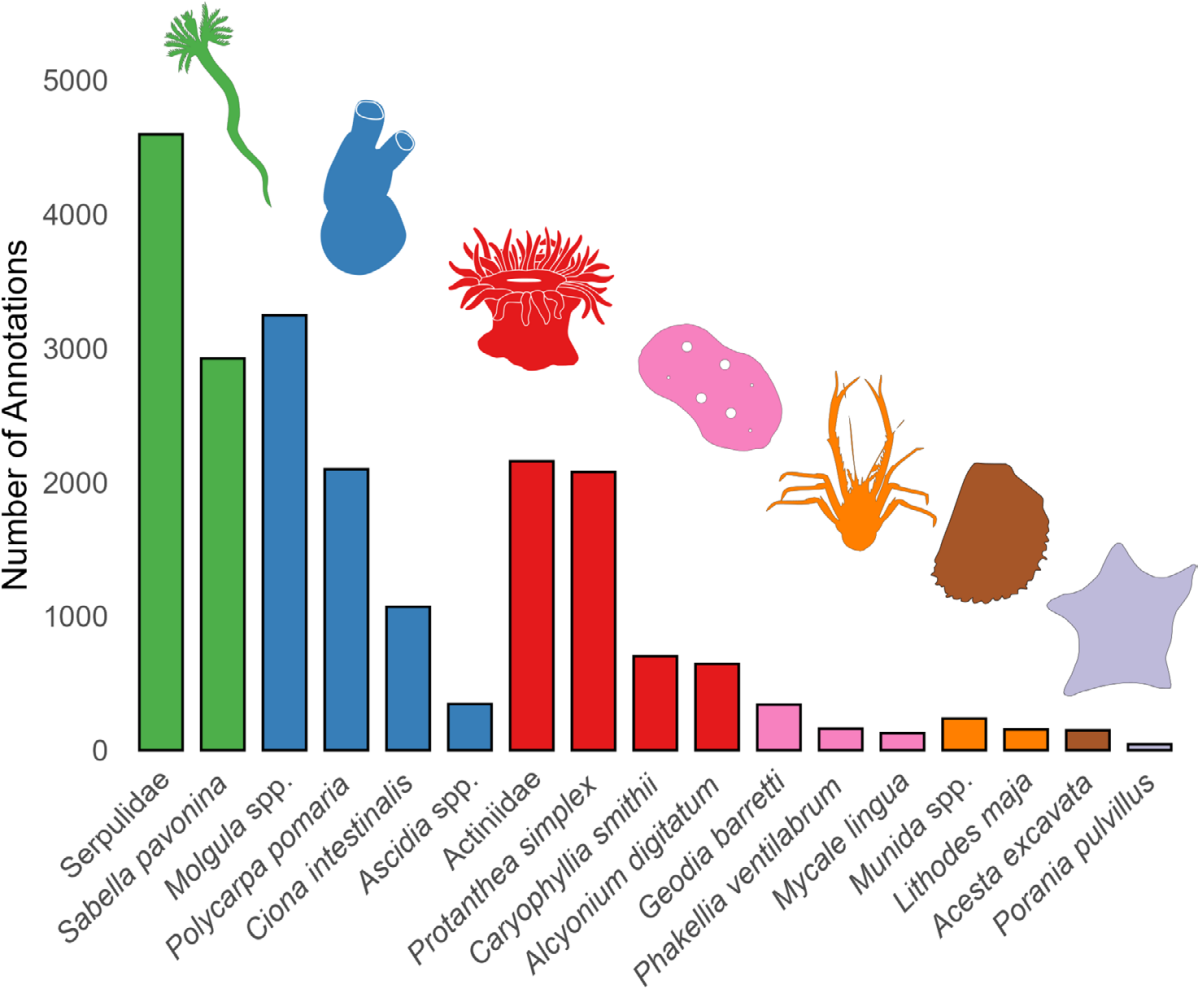
All taxa and their respective number of annotations used in the training and evaluation of the final model. Color and illustrations correspond to taxonomic affinity of investigated taxa. Green, Polychaeta (Annelida); Blue, Ascidiacea (Chordata); Red, Anthozoa (Cnidaria); Pink, Demospongiae (Porifera); Orange, Crustacea (Arthropoda); Brown, Bivalvia (Mollusca); Lavender, Asteroidea (Echinodermata).

The model was trained and evaluated on 1879 annotated image frames. A total of 627 frames with fauna were gathered from SUBSIM's image archives and an additional 1252 frames were randomly extracted from videos of seven investigated transects (Table A1) using SUBSIM Notebook “Upload clips or frames to Zooniverse”^2^. SUBSIM connects to Zooniverse^3^ which was used as the annotation platform for all frames. Prominent fauna in image frames were identified to the lowest possible taxon and annotated by drawing a bounding box over each specimen. A complete view in frame was required for each specimen visible, with identifying traits clearly visible, together with a maximum cap of 10 annotations per taxon. An 80/20% split between training/evaluation (test) datasets provided a total of 16,866 annotations for model training and 4216 for evaluation distributed across 17 taxa ranging from species to family level (Figures 2, 3).

In this study, we utilized an “off‐the‐shelf” YOLOv8 (Jocher et al. 2023) model, pretrained on the COCO2017 dataset of annotated images for object‐detection models. YOLOv8 has displayed stronger performance for fisheries monitoring when compared to commonly used Faster R‐CNN architectures (Ezzeddini et al. 2024). Furthermore, SUBSIM is intended for future real‐time detections from remote device deployments, making the faster processing time of YOLO architectures crucial to SUBSIM's infrastructure. Therefore, a YOLOv8 object‐detection model was trained on the annotated frames in SUBSIM notebook “Train models”^4^. Parameters used were a batch size of 8, 80 epochs, and an image width/height of 640/640 pixels. The final model (available on Zenodo (Nilsson, Germishuys, et al. 2024)) was applied to uploaded footage with a confidence threshold of 60% in SUBSIM notebook “Evaluate models”^5^. The model was evaluated with Receiver Operating Characteristics (ROC) metrics of recall (proportion of ground truths detected by the model; Equation 1), precision (proportion of correct model detections; Equation 2), and F1 (harmonic mean of precision and recall; Equation 3) in relation to different confidence levels of model detections. Model performance for each taxon was registered in Average Precision (AP) across all recall values Equation (4), with the model's overall performance registered as the mean AP (mAP) of all taxa Equation 5). All model detections from the evaluation phase were summarized in a normalized confusion matrix, highlighting percental overlap between the model's predictions and manual annotations. Potential biases not documented by ROC metrics and the confusion matrix were investigated through manual review of footage processed by the model.

(1) $$\;R=\frac{\mathrm{TP}}{\mathrm{TP}+\mathrm{FN}}$$

(2) $$P=\frac{\mathrm{TP}}{\mathrm{TP}+\mathrm{FP}}$$

(3) $$\;F1=2*\frac{P*R}{P+R}$$

(4) $$\;\mathrm{AP}=\sum_{r}\left(P\left(r\right)*∆R\left(r\right)\right)$$

(5) $$\mathrm{mAP}=\frac{\sum_{i=1}^{N}{\mathrm{AP}}_{i}}{N}$$

Equations (1), (2), (3), (4), (5): Equations highlighting how investigated Receiver Operating Characteristics (ROC) are calculated. 1, Recall; 2, Precision; 3, F1; 4, Average precision; 5, Mean average precision. AP, Average precision; FN, False negatives; FP, False positives; mAP, Mean average precision; *N*, Total number of classes (*i*); *P*, Precision; *r*, A specific recall value in relation to a specific precision value; *R*, Recall; TP, True positives.

### 2.3 Data Processing

Depth information was extracted from ROV footage through the video overlay. Image frames for extraction of depth information were taken from source footage using SUBSIM notebook “Upload clips or frames to Zooniverse”^2^. Between 1,900‐2,000 image frames were extracted for automatic depth reading extraction through Optical Character Recognition (OCR) at a regular interval from each transect (amounting to one depth reading per 1–4 s depending on transect length). These frames were later manually reviewed to exclude transect sections covering incorrect habitat types (e.g., the water column or soft substrates).

Extracted frames were processed in Python Version 3.12.1 (Python Software Foundation 2023). The Pillow image manipulation package (Version 10.2.0) was used to crop images to only include the video overlay's depth value and adjusted to make numbers more readable. Depth values from cropped images were read using the Optical Character Recognition (OCR) package EasyOCR (Version 1.7.1), allowing only numeric characters and decimal points. Depths read incorrectly by OCR were manually removed in Python. Image frames which were not assigned depth values by OCR received depths by linear interpolation from the two nearest established depth values. Finally, all depth values were rounded to the nearest integer value.

Output data from the object detection model applied to footage was processed in R Version 4.3.3 (R Core Team 2025) and was then expanded to include the taxa absent in each image frame. This generated a dataset of 76,280,785 datapoints, each containing the number of detections of a taxon from an individual image frame. All image frames were assigned depth values by matching their frame number to that from the corresponding depth dataset, after which the mean count of each taxon was calculated for each depth analyzed in the corresponding transect. Data from all transects were gathered into a single Darwin Core formatted occurrence dataset containing 72,369 occurrences with max count and mean count for each taxon at every depth value of each transect. This dataset, which was used for statistical analysis, was also published to the Global Biodiversity Information Framework (GBIF) (Nilsson, Anton, et al. 2024) and integrated into the Ocean Biodiversity Information System (OBIS).

### 2.4 Statistical Analysis

Statistical analyses were carried out in R version 4.3.3 (R Core Team 2025) with the aims of: (1) Modeling depth distributions of investigated taxa and comparing to empirical observations. (2) Investigating temporal trends in ecosystem function/community structure by modeling abundance trends of individual taxa and functional groups. (3) Identifying drivers of community structure trends by testing the impact of functional traits on taxon‐specific abundance trends.

To model depth distributions of investigated taxa, relative abundances in relation to depth were used to identify median depth as well as upper and lower bounds of distributions, defined as upper/lower bounds of central 68% of detections (i.e., mean +/−1 standard deviation under a normal distribution). These depths were extracted from the 50th, 16th, and 84th percentiles of abundance (measured as average detections frame^−1^ m^−1^). Modeled depth distributions were compared to depth distributions in Sweden using a GBIF dataset containing occurrences of the same 17 taxa in Sweden (GBIF.org 2024). If a taxon contained multiple species (e.g., Serpulidae, *Ascidia*, etc.), each species suggested to reside at the study site by the Swedish biodiversity portal Artdatabanken (Artdatabanken 2025a) was included in the GBIF dataset (Table A2). Data from this study was filtered out from the GBIF dataset using the “datasetKey” identifier. If the “individualCount” column of an occurrence had an NA value, the occurrence was given an “individualCount” of 1. When multiple occurrences were present at the same depth, this depth value would be repeated as many times as the corresponding occurrences. From this dataset, median depth and upper/lower bounds of distributions were extracted to represent each taxon's national distribution.

Ecosystem function was investigated through functional traits of size, lifestyle, and temperature preference, which were assigned to each taxon. Temporal abundance trends in these traits can be used as a proxy to describe whether the community is becoming more or less sensitive to temperature and fishing. Abundances of cold‐loving and large‐bodied taxa are assumed to decline under warming conditions (Burrows et al. 2019; Ohlberger 2013; Peck et al. 2009). However, abundances of large taxa could also increase following fishing restrictions as they often are slow‐growing and therefore would benefit more from the reduced disturbances. Reduced resuspended sediments from fishery regulations are expected to increase abundances of suspension feeders but would also decrease abundances of scavengers by reducing their food availability (González‐Irusta et al. 2018; Hinz et al. 2021). Furthermore, by investigating how well these traits explain taxon‐specific abundance trends, the relative impact these traits have on community‐wide abundance trends can be investigated.

Traits such as pelagic larval duration, lifespan, and reproductive frequency can also represent trawling sensitivity (González‐Irusta et al. 2018; Hinz et al. 2021). However, information on these traits was scarcely distributed across multiple databases and never covered all investigated taxa. Detailed size information was available through WoRMS Editorial Board (2025), but only regarding length. As morphologies varied greatly between taxa, length alone would not sufficiently discern large taxa from small taxa. Therefore, binary categories were used to represent size (Large—non‐polychaetes > 20 cm, Small—remaining fauna). Lifestyle was also represented through binary categories (sessile suspension feeders & mobile scavengers), based on expert judgment.

Temperature preference was represented through median temperature, which was estimated from worldwide GBIF occurrences from 2000 to 2019 of species thought to reside at the study site (GBIF.org 2025), intersected with decadal mean seafloor temperature data (at mean depth of the corresponding grid cell) from Assis et al. (2024). Occurrences from raster cells with an internal temperature difference > 2°C between minimum and maximum depth were removed together with occurrences containing a coordinate uncertainty of > 100 m and occurrences that were > 3000 km away from their nearest neighbor. Sampling bias from this dataset was reduced by randomly selecting from the remaining occurrences and removing any occurrences within a 1 km radius. From this filtered dataset (Supporting Information set Data S1), median temperature was extracted for each taxon, from which temperature preference trait categories were assigned (low, 5°C–8°C; medium, 9°C–11°C; and high, 11°C–12°C).

To investigate changes in community structure and ecosystem function over time (year), a generalized linear model (GLM) was applied to scaled abundances of individual taxa and trait categories. Variation in density caused by seasonality and depth was controlled for by including day of year (day), day of year squared (day^2^), depth, and square root of depth (√depth) (all parameters scaled) in the model. To investigate drivers behind temporal changes in community structure, statistical tests were made on how traits impact taxon‐specific abundance coefficients. For the binary size and lifestyle trait categories, Wilcoxon rank‐sum tests were applied. For the continuous median preferred temperature, a linear regression was utilized. Thus, we were able to analyze both temporal abundance trends of individual taxa and traits as well as relationships between traits and the abundance trends of all investigated taxa.

## 3 Results

### 3.1 Model Performance

**FIGURE 4:**
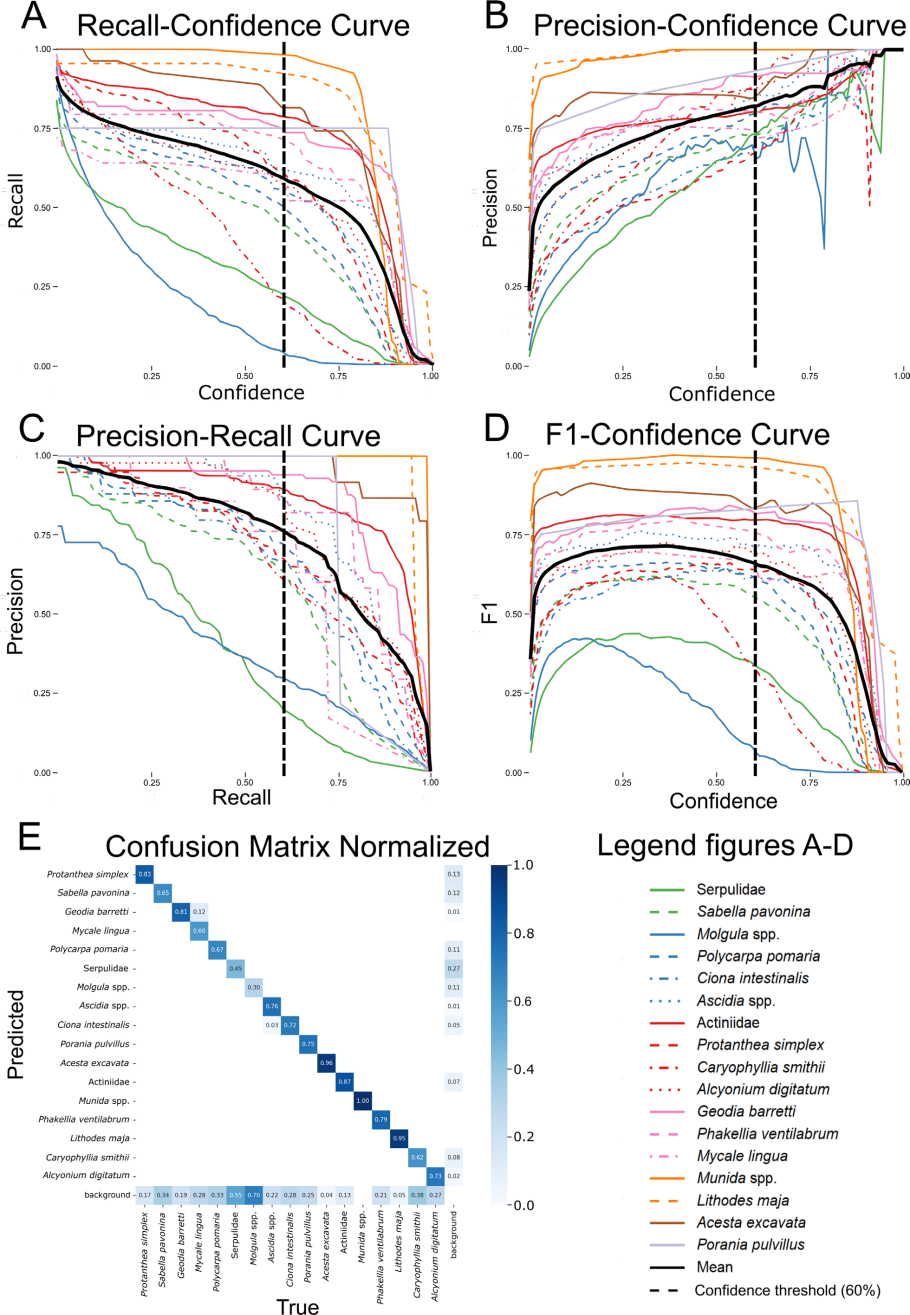
Receiver Operating Characteristics (ROC) plots and confusion matrix from model evaluation. (A) Recall‐confidence curve. (B) Precision‐confidence curve. (C) Precision‐recall curve. (D) F1‐confidence curve. (E) Normalized confusion matrix.

The training dataset (Figure 3) produced an object‐detection model for 17 invertebrate taxa with a mean Average Precision (mAP) score of 0.738. High variation in model performance was found between taxa (Figure 4). Larger taxa with distinct features displayed higher receiver operating characteristics (ROC) scores, while smaller, less distinct taxa had lower ROC scores (Figures 2 and 4). Recall (Figure 4A) being lower than precision (Figure 4B,C) and the confusion matrix (Figure 4E) both suggest that false negative detections were more common than false positives. The model was unable to detect all occurrences of certain taxa in the evaluation dataset, highlighted by recall values not reaching 1 at a confidence level of 0 (Figure 4A). However, precision metrics highlight that the model was able to exclude false positives from all taxa at confidence levels > 0.972 (Figure 4B). From the precision‐recall curve (Figure 4C), an Average Precision (AP) score was extracted for each taxon (Supporting Information set S2), from which the mAP score across all taxa was documented at 0.738, indicating that detections made by the model were rarely false (although this varied between taxa). Mean F1 peaked at a confidence level of 0.355 (Figure 4D), indicating a confidence threshold that would ensure the highest number of correct predictions with the lowest degree of false predictions. However, this confidence threshold was disregarded in favor of a 60% confidence threshold to reduce influence from false positives.

**FIGURE A3:**
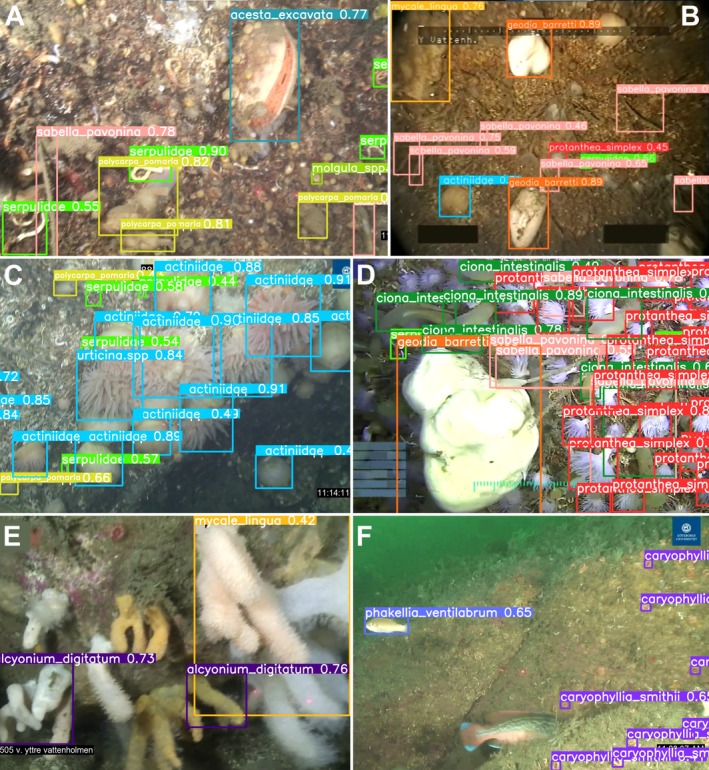
Snapshots of the object‐detection model annotating fauna from archived footage of the study site. (A, B) Deep habitats. (C, D) Intermediate‐deep habitats. (E) *Alcyonium digitatum* at shallow depths, one incorrectly annotated as *Mycale lingua* . (F) *Caryophyllia smithii* at intermediate‐shallow depths with a fish incorrectly annotated as *Phakellia ventilabrum* .

The application of the object detection model (Nilsson, Germishuys, et al. 2024) to the selected footage resulted in a 72,369‐occurrence dataset (Nilsson, Anton, et al. 2024). Manual review of model detections on footage (Figure A3A–D in Appendix A) corroborates results from model training (Figure 4) indicating that detections are generally true, with a higher proportion of false negatives toward small or indistinct taxa. Manual review also showed repeated cases of false positives between morphologically similar taxa and on objects/organisms not included in model training (Figure A3E,F in Appendix A). As this was not highlighted in the confusion matrix (Figure 4E), the model may be overfitted to its training data, highlighting a potential lack of variation in the evaluation dataset.

### 3.2 Depth Distributions

**FIGURE 5:**
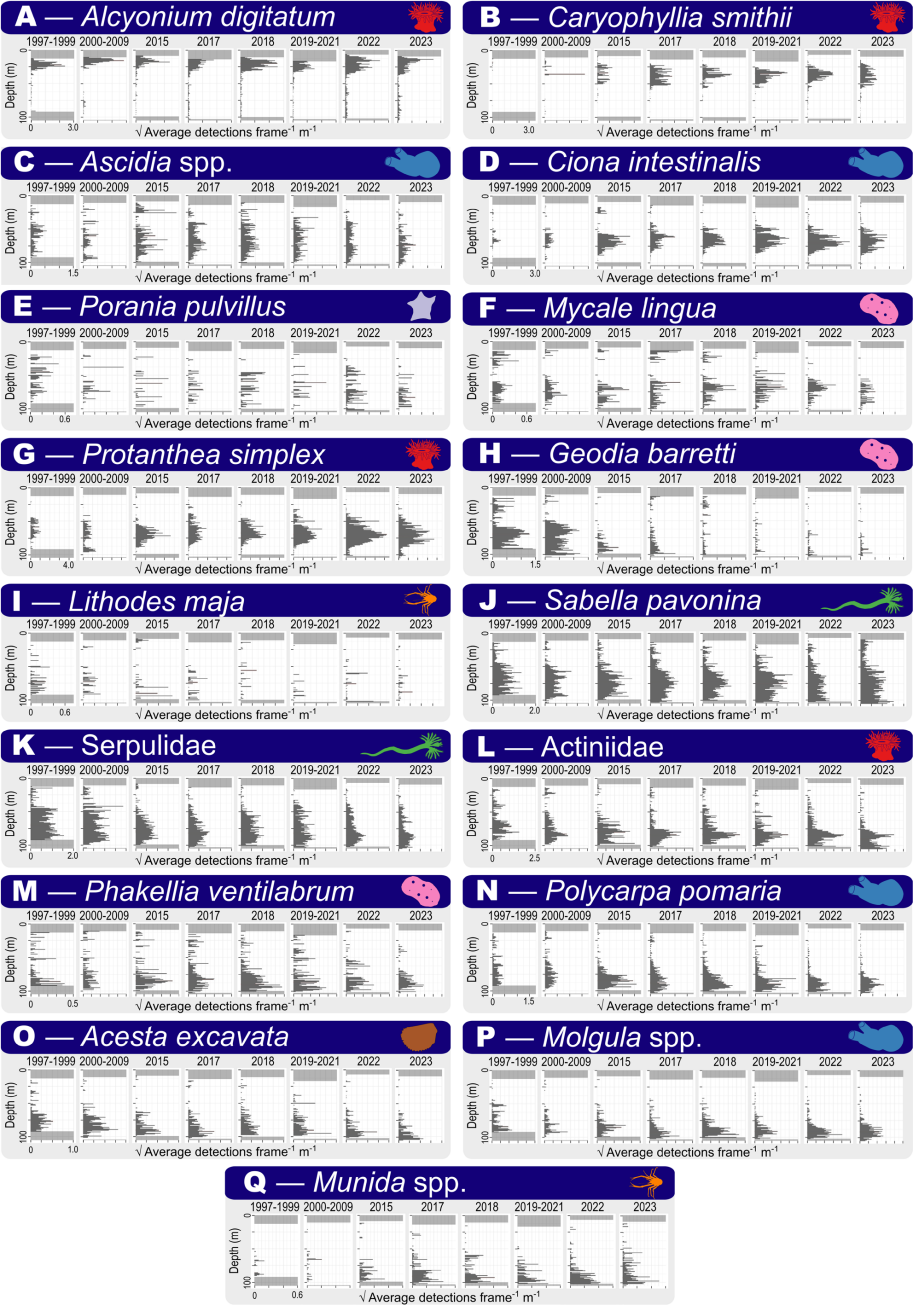
Vertical histograms of square‐root transformed abundance (average detections frame^−1^ m^−1^) of each taxon. Detections from each time‐period were grouped for visualization purposes. Kosterhavet National Park was inaugurated in 2009. Gray fields highlight depths not covered. Number of transects in each time‐period: 1997–1999 = 6, 2000–2009 = 10, 2015 = 5, 2017 = 13, 2018 = 8, 2019–2021 = 8, 2022 = 11, 2023 = 9.

Model‐based observations provide a continuous spatiotemporal abundance profile of the rock wall community (Figure 5). Vertical distributions were generally unimodal, displaying each taxon's optimal depth range. The shallowest depths of the study site are dominated by *Alcyonium digitatum* , followed by *Caryophyllia smithii* below (Figure 5A,B). A distinct vertical zonation structure is seen at these depths as distribution ranges of neither *Alcyonium digitatum* , nor *Caryophyllia smithii* overlap with any other taxa (Table 1). Sharp increases in abundance of remaining fauna can be seen at depths of ~50 m (Figure 5C–Q), corroborated by median depth of these taxa ranging from 67 to 94 m (Table 1). Amongst these 15 taxa, some vertical structure is also likely present, albeit to a lesser degree, with several overlapping distributions (Table 1). Here, median depths of *Protanthea simplex* , *Geodia barretti*, *Mycale lingua* , *Ascidia* spp., *Ciona intestinalis* , and *Porania pulvillus* were found between 67 and 71 m, while median depths of *Sabella pavonina* , *Polycarpa pomaria* , Serpulidae, *Molgula* spp., *Acesta excavata* , Actiniidae, *Munida* spp., *Phakellia ventilabrum* , and *Lithodes maja* ranged between 77 and 94 m (Table 1).

**TABLE 1:** Table of modeled and national depth distributions, rounded to nearest integer. Depth distribution includes median depth and lower/upper bounds of the central 68% of detections/occurrences. Presented as “median (lower‐upper).”

	Modeled distribution (m)	National distribution (m)
*Alcyonium digitatum*	17 (13–26)	27 (24–32)
*Caryophyllia smithii*	36 (30–43)	32 (25–50)
*Ascidia* spp.	67 (45–82)	24 (19–38)
*Ciona intestinalis*	67 (58–73)	5 (2–19)
*Porania pulvillus*	68 (44–88)	48 (28–57)
*Mycale lingua*	69 (57–83)	198 (159–251)
*Protanthea simplex*	70 (60–79)	30 (5–80)
*Geodia barretti*	71 (62–93)	92 (67–165)
*Lithodes maja*	77 (57–97)	138 (33–242)
*Sabella pavonina*	78 (62–99)	88 (30–135)
Serpulidae	81 (60–92)	26 (0–104)
Actiniidae	85 (78–98)	29 (18–252)
*Phakellia ventilabrum*	87 (74–94)	278 (182–333)
*Polycarpa pomaria*	89 (80–95)	159 (114–203)
*Acesta excavata*	90 (77–99)	85 (56–176)
*Molgula* spp.	92 (85–98)	13 (5–29)
*Munida* spp.	94 (83–100)	148 (53–274)

Out of the 17 depth distributions modeled in this study, 11 overlapped with their corresponding national depth distributions (Table 1). From the six taxa that were observed outside their national depth range, three were observed at greater depths in this study (*Molgula* spp., *Ascidia* spp., *Ciona intestinalis* ), and three were found in more shallow depths ( *Mycale lingua* , *Polycarpa pomaria* , *Phakellia ventilabrum* ). Estimates of national depth distributions for *Geodia barretti*, *Polycarpa pomaria* , and *Molgula* spp. are, however, uncertain as they have ≤ 10 occurrences (Table A2).

### 3.3 Temporal Trends and Their Drivers

Results from general linear models (GLMs) on the abundance of each taxon (Table 2) highlight a changing community structure, with time (year) having a significant (*p* < 0.05) impact on the abundance of 13 of the 17 investigated taxa (Table 2). The four taxa without significant trends were: *Mycale lingua* , *Polycarpa pomaria* , *Molgula* spp., and *Phakellia ventilabrum* . From the 13 taxa with significant trends, the eight taxa: *Protanthea simplex* , *Sabella pavonina* , *Ascidia* spp., *Ciona intestinalis* , Actiniidae, *Munida* spp., *Caryophyllia smithii* , and *Alcyonium digitatum* displayed significantly increasing abundances. The remaining five taxa with significantly decreasing abundances included: *Geodia barretti*, Serpulidae, *Porania pulvillus* , *Acesta excavata* , and *Lithodes maja* . Additionally, *Molgula* spp. displayed a near‐significant increase in abundance (*p* = 0.062).

**TABLE 2:** Temporal changes in taxon abundance from general linear models (GLMs) on abundance (average detections frame^−1^ m^−1^) of taxa and relevant traits in relation to year, square root of depth (√depth), depth, day, and day squared (day^2^) (all scaled). Presented as “Estimate (standard error)” significant values are marked with *p < 0.05, **p < 0.01, and ***p < 0.001.

	*Alcyonium digitatum*	*Caryophyllia smithii*	*Ascidia* spp.	*Ciona intestinalis*
Year	0.04 (0.01)**	0.09 (0.02)***	0.04 (0.02)**	0.12 (0.02)***
√Depth	−2.91 (0.11)***	0.60 (0.12)***	0.77 (0.13)***	1.43 (0.12)***
Depth	2.56 (0.11)***	−0.81 (0.12)***	−0.68 (0.13)***	−1.35 (0.12)***
Day	−0.09 (0.06)	0.09 (0.07)	0.15 (0.07)*	0.17 (0.07)*
Day^2^	0.13 (0.07)	−0.11 (0.07)	−0.10 (0.07)	0.21 (0.07)**

	*Porania pulvillus*	*Mycale lingua*	*Protanthea simplex*	*Geodia barretti*
Year	−0.06 (0.02)***	−0.02 (0.02)	0.16 (0.02)***	−0.44 (0.01)***
√Depth	−0.31 (0.13)*	0.38 (0.13)**	1.24 (0.12)***	0.09 (0.11)
Depth	−0.28 (0.13)*	−0.30 (0.13)*	−1.10 (0.12)***	0.02 (0.11)
Day	0.12 (0.08)	0.15 (0.07)*	0.13 (0.07)	0.19 (0.07)**
Day^2^	−0.10 (0.08)	0.15 (0.08)*	−0.21 (0.07)**	−0.24 (0.07)***

	*Lithodes maja*	*Sabella pavonina*	Serpulidae	Actiniidae
Year	−0.05 (0.02)*	0.03 (0.02)*	−0.23 (0.01)***	0.03 (0.02)*
√Depth	−0.066 (0.13)	0.17 (0.12)	0.40 (0.12)***	−0.99 (0.12)***
Depth	0.12 (0.13)	0.17 (0.12)	0.76 (0.12)***	1.30 (0.12)***
Day	0.06 (0.08)	0.17 (0.07)*	0.11 (0.07)	0.20 (0.07)**
Day^2^	−0.07 (0.08)	−0.17 (0.07)*	−0.13 (0.07)	−0.19 (0.07)**

	*Phakellia ventilabrum*	*Polycarpa pomaria*	*Acesta excavata*	*Molgula* spp.
Year	< 0.01 (0.01)	0.02 (0.02)	−0.09 (0.02)***	0.03 (0.02)
√Depth	−1.03 (0.12)***	−1.45 (0.12)***	−1.12 (0.12)***	−1.44 (0.12)***
Depth	1.25 (0.12)***	1.78 (0.12)***	1.37 (0.12)***	1.69 (0.12)***
Day	0.13 (0.07)	−0.24 (0.07)***	−0.02 (0.07)	0.11 (0.07)
Day^2^	−0.14 (0.07)	−0.24 (0.07)***	−0.01 (0.07)	−0.15 (0.07)*

	*Munida* spp.			
Year	0.07 (0.02)***			
√Depth	−1.23 (0.12)***			
Depth	1.45 (0.12)***			
Day	0.02 (0.07)			
Day^2^	−0.01 (0.07)			

Changes in community structure were also visible through changes in ecosystem function, with significant trends in six of the seven trait categories (Table 3). Significant trends were observed in both size categories, with abundances increasing in small fauna and decreasing in large fauna. Sessile suspension feeders displayed a significant increase while mobile scavengers displayed the only non‐significant trend. Low‐temperature adapted taxa were declining while taxa adapted to medium and high temperatures increased over time.

**TABLE 3:** Temporal changes in ecosystem function from general linear models (GLMs) on abundance (average detectionss frame^−1^ m^−1^) of taxa and relevant traits in relation to year, square root of depth (√depth), depth, day, and day squared (day^2^) (all scaled). Presented as “Estimate (standard error)” significant values are marked with *p < 0.05, **p < 0.01, and ***p < 0.001.

	Size (Small)	Size (Large)	Lifestyle (Sessile suspension feeders)	Lifestyle (Mobile scavengers)
Year	0.06 (< 0.01)***	−0.02 (0.01)***	0.04 (< 0.01)***	−0.01 (0.01)
√Depth	0.51 (0.04)***	−0.83 (0.05)***	0.21 (0.03)***	−0.32 (0.07)***
Depth	−0.44 (0.04)***	0.87 (0.05)***	−0.13 (0.03)***	0.42 (0.07)***
Day	0.09 (0.02)***	0.06 (0.03)*	0.08 (0.02)***	0.07 (0.04)
Day^2^	−0.12 (0.02)***	−0.05 (0.03)*	−0.10 (0.02)***	−0.06 (0.04)

	Median temperature (Low)	Median temperature (Medium)	Median temperature (High)
Year	−0.22 (0.01)***	0.05 (0.01)***	0.06 (0.01)***
√Depth	−0.14 (0.06)*	0.33 (0.05)***	0.06 (0.05)
Depth	0.25 (0.06)***	−0.23 (0.05)***	−0.04 (0.05)
Day	0.10 (0.04)**	0.08 (0.03)*	0.09 (0.03)***
Day^2^	−0.13 (0.04)***	−0.11 (0.03)***	−0.10 (0.03)***

**FIGURE 6:**
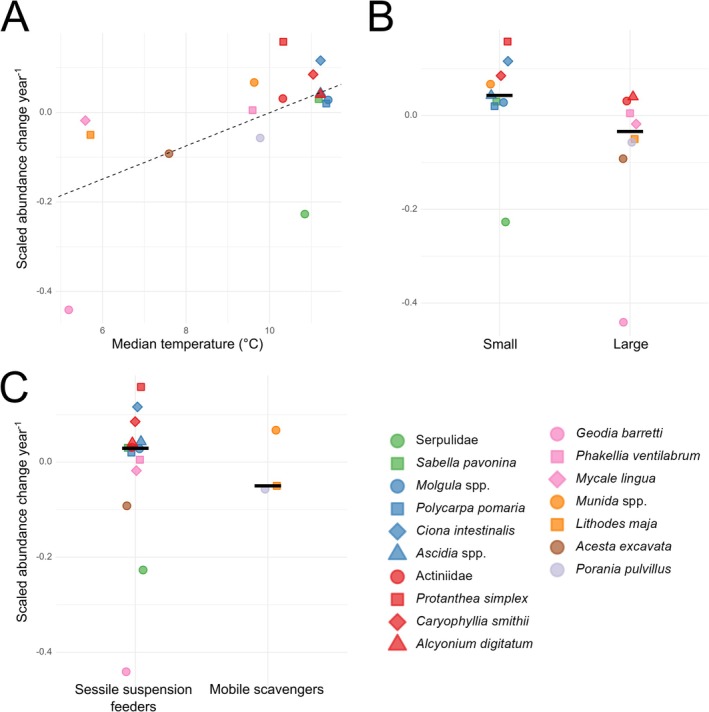
Identification of potential drivers behind abundance changes. Scatter plots of taxon‐specific abundance change per year (Table 2) plotted against potential drivers. (A) Median temperature as a driver behind abundance change (estimate = 0.578, std. error = 0.211; r = 0.578, *p* = 0.0151), dashed line represents regression line. (B) Size as a driver behind abundance change (*p* = 0.0274). (C) Lifestyle as a driver behind abundance change (*p* = 0.676). Black bars in Figures (B, C) highlight each group's medians.

A clear correlation was found between median temperatures of investigated taxa and their abundance coefficients (*r* = 0.580, *p* = 0.015, Figure 6A). Size as a predictor for taxon‐specific abundance coefficients showed a significant (*p* = 0.027, Figure 6B) impact. Here, the majority of large taxa declined, while all small taxa except Serpulidae displayed increasing abundances. These trends suggest that preferred temperature and size have acted as strong predictors of taxon‐specific changes in abundance over time. The overall abundance of sessile suspension feeders increased (Table 3); however, lifestyle served as a poor predictor of abundance trends of individual taxa (Figure 6C) as both sessile suspension feeders and mobile scavengers had large spreads in their abundance coefficients. This further highlights that lifestyle alone was not a driver behind the observed trends in community structure.

## 4 Discussion

### 4.1 Model Performance

Results from the model training output and subsequent manual review (Figures 4 and A3A–D) indicate that the model can reliably identify the 17 invertebrate taxa we analyzed. This is corroborated by the output data repeatedly showing unimodal depth distributions at similar depths for all taxa (Figure 5) which generally aligned with empirical observations from Sweden (Table 1). However, the model is limited to the 17 taxa with sufficient annotations to warrant inclusion in the model. This causes the omission of the “long tail” of rare taxa occurring at the site, leading to a limited view of the investigated community (Villon et al. 2022). Ecological applications of computer vision are also limited by the same constraints as manual video‐based monitoring, e.g., key morphological characteristics not being visible in footage, forcing the use of higher taxonomic ranks (e.g., Table A2) or morphotypes, leading to a loss of community complexity (Hanafi‐Portier et al. 2021). Thus, computer vision and deep learning for ecological monitoring cannot be viewed as a replacement for physical sampling and taxonomic investigations, but should instead be viewed as a complement capable of collecting vast volumes of data for specific taxa, crucial for large‐scale spatiotemporal monitoring.

While variation in the model's performance for different taxa (Figure 4) makes comparison of abundances between taxa challenging, the data is well suited for investigations of each taxon individually. However, at shallow depths certain taxa were incorrectly annotated or annotated on non‐modeled taxa/objects (e.g., Figure A3E,F), leading to false shallow abundance peaks (Figure 5F,H,L,M). This is likely caused by what Villon et al. (2022) describe as the “open world problem” for ecological applications of computer vision models. This problem states that the model will encounter environmental conditions (e.g., turbidity/lighting) and objects/species not encountered in its training. Since most taxa resided at deep sections of the study site, the majority of training frames (and thus, also annotations; Figure 3) were taken from deep habitats where the ROV's lights were on. This may have excluded variation at shallow depths (where the ROV's lights were off) that was not accounted for in training/evaluation datasets, reducing the model's confidence under these lighting conditions without it being reflected in the model's evaluation (Figure 4). The visual setting from which training frames are taken also influences model accuracy (Lürig et al. 2021). Therefore, curating training/evaluation datasets to include more variation (e.g., more shallow image frames) would strengthen the model's performance and facilitate the identification of any model limitations (e.g., missing false positives from the confusion matrix; Figure 4E).

Further support for modeled detections can also be found when comparing modeled findings to previous studies. A benthic biodiversity report from the Kosterhavet National Park by Jonsson (2014) manually documents all taxa investigated in our study at depths comparable to where modeled detections were made. Modeled results also matched previously documented temporal trends, such as the decline of *Geodia barretti* in the Kosterhavet Marine Protected Area (MPA) in the late 2000s (Guihen et al. 2012; Figure 5H) and an emergence of *Caryophyllia smithii* in Sweden in the early 2000s (Obst et al. 2018; Figure 5B). Thus, the model is capable of reproducing results from independent studies, supporting the validity of distributions/trends documented in this study as well as providing insight into previously undocumented trends and distributions. Furthermore, the immense volume of data collected enables a high‐scale/resolution quantification of trends and distributions which was previously not possible, opening up for comparative analyses across depth and time within individual taxa, as well as correlative analyses with potential drivers.

### 4.2 Depth Distributions

Overview of vertical distributions shows clear differences in depth ranges and abundances of the taxa investigated at the study site (Figure 5). Few taxa were found at shallow depths. Only *Alcyonium digitatum* and *Caryophyllia smithii* had their modeled median depth at depths < 40 m (17 and 36 m respectively), while the remaining 15 taxa had median depths distributed between 67 and 94 m (Table 1). A lower abundance of the investigated taxa can be expected in shallow waters as sessile invertebrates face higher spatial competition with algae in more well‐lit habitats (Schaefer et al. 2024). Most of the investigated taxa instead displayed sharp increases in abundance at ~50 m depth, where the substrate transitions from a slope to a wall (Figure 5D,F–Q). The drastic change in community structure was likely observed due to the steep angle of inclination reducing light influx, but also sedimentation, allowing the sessile invertebrates to conduct more unobstructed suspension feeding (Cárdenas and Montiel 2015; Miller and Etter 2011). This is further supported by repeated findings of deeper‐dwelling taxa ( *Protanthea simplex* , *Ciona intestinalis* , *Sabella pavonina* , Serpulidae, *Ascidia* spp.) attached to vertical surfaces at more shallow depths than the central 68% of their distribution range (Table 1). Thus, these findings suggest that for most investigated taxa, angle of inclination may be a more limiting factor than depth.

Modeled depth distributions generally occurred at the same depths as their reported occurrences in Sweden. However, the central 68% of detections/occurrences for six taxa had no overlap (Table 1; the model suggests deeper distributions than empirical observations for *Ascidia* spp., *Ciona intestinalis* , and *Molgula* spp. and shallower for *Mycale lingua* , *Polycarpa pomaria* , *Phakellia ventilabrum* ). Open‐access biodiversity data display an exponential decline in occurrences with increased depth, leading to potential data gaps from deeper habitats (Bridges and Howell 2025) which this study could address by expanding the depth range deeper for some taxa. Furthermore, it is also possible to expand distributions of deep‐dwelling taxa to shallower depths as the Koster Fjord is heavily influenced by influxes of deep Atlantic water via the Norwegian trench (Polovodova Asteman et al. 2021). This has enabled deep‐dwelling species to reach unusually shallow depths in the MPA (Tullroth et al. 2019; Wisshak et al. 2005), when compared to the rest of Sweden. However, modeled depth distributions may also differ from occurrence‐based distributions due to model biases (i.e., the open world problem *sensu* Villon et al. (2022)) and potential mismatches in abundance proportions of multispecies taxa (Table A2). Nonetheless, we consider this study's repeated detections of taxa outside their previously reported depth ranges as sufficient grounds for updating their depth ranges in Sweden, especially for taxa with few occurrences (Table A2).

The modeled community structure from this study showcases the area's rich community of benthic invertebrates, especially where depth and angle of inclination increase. These findings align with previous investigations suggesting that vertical rock walls act as biodiversity hotspots and sites of refuge for benthic invertebrates (Gasbarro et al. 2018; Miller and Etter 2011; Robert et al. 2015, 2017). Furthermore, the changing community composition along the study site's depth gradient highlights how large depth gradients can serve to heighten local biodiversity.

### 4.3 Temporal Trends and Their Drivers

Analysis of temporal trends in abundance (Tables 2 and 3) shows that the study site is a dynamic environment, with several ongoing trends in community structure and ecosystem function. Out of the 17 investigated taxa, 13 displayed significant changes in abundance (eight positive, five negative; Table 2). The observed changes in taxon abundance are also reflected in a changing ecosystem function, with the investigated community shifting toward a regime of smaller, more heat‐tolerant suspension feeders (Table 3). However, some abundance trends may remain undocumented (e.g., potentially missed shallow occurrences of *Ascidia* spp. and *Ciona intestinalis* ) or be incorrect. One potentially skewed trend could be the sharp decrease in abundance observed for Serpulidae (Table 2), which generally grow close to the substrate (Figure 2C,F). This decline occurs primarily at depths where *Protanthea simplex* increases (Figure 5G,K), which can grow in dense assemblages that can obscure the bedrock (Figure 2B,C). The increased abundance of *Protanthea simplex* may thus have obscured Serpulid worms from view.

When investigating the suitability of chosen traits as predictors for taxon‐specific abundance change, temperature had the strongest correlation (Figure 6) with colder preferred temperatures being associated with decreasing abundances (Table 3). The strongest reduction in abundance was seen in *Geodia barretti*, which had the coldest preferred temperature (Figure 6A). This large, habitat‐forming sponge is now virtually extinct from the study site (Figure 5H), a loss that was documented by Guihen et al. (2012) in the form of mass die‐off events between 2006 and 2009, thought to be caused by heat waves in 2006 and 2008. Another large, habitat‐forming cold‐water species that displayed a sharp decline was *Acesta excavata* (Table 2, Figures 5O and 6A). This species has displayed a limited capacity for physiological responses to stressors of heat and sedimentation (Scanes et al. 2024). Thus, the decline of *Geodia barretti* and *Acesta excavata* highlights that the observed warming in the Koster area (Figure A1 in Appendix A) may not only reduce the abundance of cold‐water species, but also the availability of biogenic habitats.

Abundance trends of investigated size classes showed increases in small taxa and reductions in large taxa that were both significant (Table 3). Size was another strong predictor of abundance trends at the taxon level as the Wilcoxon rank‐sum test on size classes yielded significant results (Figure 6B). This conflicts with the assumption that reduced trawling near the study site would benefit the larger taxa (Hinz et al. 2021) since there would be less disturbances from sedimentation. However, the parallel effect of increasing temperatures (Figure A1 in Appendix A) may act as a counteracting force because increased temperatures generally benefit smaller body sizes (Ohlberger 2013). Thus, even though size may be an additional predictor of taxon‐specific abundance change, its apparent relation to temperature reduces the insight into the community's response to reduced trawling.

As lifestyle (mobility & feeding mode) has less of a direct connection to climate, it serves as a better indicator for community response to reduced trawling. While the community responds as expected (González‐Irusta et al. 2018) with a significant increase in sessile suspension feeders (Table 3), lifestyle served as a poor predictor for taxon‐specific changes (Figure 6C). This result is likely influenced by a low number of mobile scavenging taxa investigated and factors such as temperature preference and size being stronger drivers of observed abundance trends. However, it is still possible that trawling regulations could contribute to the many increasing abundances documented in this study.

### 4.4 Implications for Management

Our study shows that marine rock walls with large depth gradients are important targets for protection in MPAs. Rock walls are less susceptible to physical disturbance (Robert et al. 2017), while steep depth gradients facilitate benthic vertical migration in response to climate change (Pinsky et al. 2020). The diverse communities at the study site (Figure 2), display widespread trends of increasing abundances for trawling‐sensitive taxa ( *Protanthea simplex* —NT (Artdatabanken 2025c), *Munida rugosa/sarsi*—NT/VU (Artdatabanken 2025b); *Protanthea simplex* , *Sabella pavonina* , *Ascidia* spp., *Ciona intestinalis* , and *Urticina eques* (Actiniidae) highlighted by Jonsson (2014) as sensitive), highlighting the efficacy of the MPA's reduced trawling activities.

Despite widespread positive trends throughout the timeframe studied, abundances of the key habitat‐forming species *Geodia barretti* and *Acesta excavata* (Table 2) are still in decline. Their remaining populations are concentrated in the deepest sections of the study site (Figure 5H,O), likely due to their temperature sensitivity (Guihen et al. 2012; Scanes et al. 2024; Figure 6C). As they are also sensitive to sedimentation (Kutti et al. 2015; Scanes et al. 2024), remaining populations of *Geodia barretti* and *Acesta excavata* are likely further disadvantaged by the continued trawling at > 60 m depth (Tullroth et al. 2019). Therefore, to support deepening populations of vulnerable, yet ecologically important species, we recommend an expansion of the trawling ban to cover all depths of the MPA.

As temperatures are expected to rise further (Calvin et al. 2023), a future complete loss of cold‐water species in the MPA could also be expected. Hence, successful preservation of temperature‐sensitive species may only be achieved by the establishment of new marine protected areas in the region. Through the establishment of protected areas combined with active restoration efforts in deeper, colder waters, it is possible to preserve populations of key cold‐water species, ensuring a continuation of the habitats they form.

Our study demonstrates that continuous monitoring with video technology provides invaluable data and opportunities to understand and quantify long‐term functional responses of complex communities to multiple anthropogenic drivers. Training deep‐learning algorithms to detect key sensitive and/or ecologically significant indicator taxa in footage will also enable information on ecosystem status to be collected in a fast and cost‐efficient manner. Such knowledge is critical in management of marine ecosystems and can be obtained by systematic video monitoring programs across a range of key habitats. We therefore recommend standardized and systematic video monitoring, coupled with deep‐learning algorithms for use in MPA monitoring and management.

## Author Contributions

**Christian L. Nilsson:** conceptualization (equal), data curation (lead), formal analysis (lead), methodology (supporting), project administration (equal), validation (lead), visualization (lead), writing – original draft (lead). **Søren Faurby:** formal analysis (equal), methodology (equal), project administration (supporting), supervision (lead), writing – review and editing (lead). **Emil Burman:** formal analysis (supporting), software (equal), supervision (equal), writing – review and editing (equal). **Jurie Germishuys:** data curation (supporting), resources (supporting), software (lead), writing – review and editing (equal). **Matthias Obst:** conceptualization (equal), data curation (supporting), funding acquisition (lead), methodology (supporting), project administration (lead), resources (equal), software (supporting), supervision (lead), writing – original draft (supporting).

## Conflicts of Interest

The authors declare no conflicts of interest.

## Supporting information


**Data S1:** ece372091‐sup‐0001‐DataS1.xlsx.

**Data S2:** ece372091‐sup‐0002‐DataS2.xlsx.

## Data Availability

The object‐detection model used for detecting the investigated taxa is publicly available on Zenodo (Nilsson, Germishuys, et al. (2024), https://doi.org/10.5281/zenodo.13589902). The datasets used for all analyses are publicly available (Nilsson, Anton, et al. (2024), https://doi.org/10.15468/rzhmef; GBIF.org (2024), https://doi.org/10.15468/dl.rcne77; GBIF.org (2025) https://doi.org/10.15468/dl.azec6t; Assis et al. (2024) https://doi.org/10.1111/geb.13813). Information not suitable for a single table is included as Supporting Information for publication. The code and datasets needed to reproduce all results from this study can be found in the GitHub repository “Koster‐Deep‐Learning‐Ecology” (https://doi.org/10.5281/zenodo.15249144).

## Endnotes

^1^

www.subsim.se.

^2^

https://github.com/ocean‐data‐factory‐sweden/kso/blob/dev/notebooks/classify/Upload_subjects_to_Zooniverse.ipynb.

^3^

www.zooniverse.org.

^4^

https://github.com/ocean‐data‐factory‐sweden/kso/blob/dev/notebooks/analyse/Train_models.ipynb.

^5^

https://github.com/ocean‐data‐factory‐sweden/kso/blob/dev/notebooks/analyse/Evaluate_models.ipynb.

## Appendix A

**TABLE A1:** All transects investigated in this study, their depths and durations. Some depths were removed due to the Remotely Operated Vehicle (ROV) departing from the investigated habitats. *Highlights transects included in annotations dataset.

Transect ID	Date	Depths covered (m)	Duration (HH:MM:SS)
970827	27‐8‐1997	50–91	00:25:29
971104	4‐11‐1997	16–63	00:52:27
990622	22‐6‐1999	59–76	00:25:48
990719	19‐7‐1999	13–80	00:50:42
990805*	5‐8‐1999	13–82	00:38:37
990806*	6‐8‐1999	26–92	00:28:32
000317	17‐3‐2000	50–87	00:39:35
000617	17‐6‐2000	50–99	00:21:39
010810	10‐8‐2001	38–62	00:08:51
011016	16‐10‐2001	38–41, 45–104	00:32:57
020819	19‐8‐2002	62–87	00:12:57
041119	19‐11‐2004	50–80	00:39:00
050531	31‐5‐2005	19–31, 67–84	00:37:50
060309	9‐3‐2006	70–83	00:19:02
090202	2‐2‐2009	28–37, 84–98	00:23:07
090812	12‐8‐2009	11–24	00:35:09
150424	24‐4‐2015	9–90	00:29:05
150812_1	12‐8‐2015	63–94	00:18:45
150812_2*	12‐8‐2015	11–99	00:34:22
150829	29‐8‐2015	24–97	00:20:57
150926*	26‐9‐2015	49–98	00:24:47
170505	5‐5‐2017	13–90	00:30:43
170714_1	14‐7‐2017	32–98	00:28:02
170714_2	14‐7‐2017	22–102	00:26:24
170728_1	28‐7‐2017	44–93	00:22:15
170728_2	28‐7‐2017	52–96	00:28:21
170808_1	8‐8‐2017	53–100	00:25:48
170808_2	8‐8‐2017	40–97	00:30:13
170811_1	11‐8‐2017	25–92	00:37:32
170811_2	11‐8‐2017	28–92	00:51:11
171011_1	11‐10‐2017	24–80	00:24:19
171011_2	11‐10‐2017	32–87	00:27:54
171013_1	13‐10‐2017	60–94	00:13:52
171013_2	13‐10‐2017	55–103	00:15:53
1805XX	May 2018 (exact date uncertain)	11–98	01:23:25
180,626	26‐6‐2018	33–96	00:25:58
180706_1	6‐7‐2018	49–96	00:31:44
180706_2	6‐7‐2018	49–99	00:35:28
180713_1	13‐7‐2018	46–98	00:41:07
180713_2	13‐7‐2018	33–100	00:37:17
180814_1	14‐8‐2018	26–95	00:33:54
180814_2	14‐8‐2018	28–99	00:31:48
190,213	13‐2‐2019	18–96	00:45:17
190812_1	12‐8‐2019	17–86	00:36:45
190812_2	12‐8‐2019	33–81	00:23:12
200910_1	10‐9‐2020	20–97	00:30:52
200910_2*	10‐9‐2020	21–103	00:36:05
210510	10‐5‐2021	62–99	02:15:45
210525	25‐5‐2021	36–100	00:25:29
210527	27‐5‐2021	40–101	00:31:54
220406	6‐4‐2022	24–100	00:47:54
220420	20‐4‐2022	35–98	00:48:08
220628_1	28‐6‐2022	18–101	01:01:00
220628_2	28‐6‐2022	12–82	00:38:08
220715_1	15‐7‐2022	31–96	00:31:45
220715_2*	15‐7‐2022	29–97	00:30:45
220805_1	5‐8‐2022	28–99	00:31:44
220805_2*	5‐8‐2022	14–102	00:38:25
220902	2‐9‐2022	11–37, 52–96	00:33:58
220921	21‐9‐2022	31–102	00:14:44
220929	29‐9‐2022	7–81	00:24:35
230502	2‐5‐2023	24–103	00:28:21
230,505	5‐5‐2023	11–104	00:32:19
230524_1	24‐5‐2023	12–105	00:43:27
230524_2	24‐5‐2023	11–97	00:28:06
230627_1	27‐6‐2023	20–97	00:28:55
230627_2	27‐6‐2023	24–97	00:49:15
230811_1	11‐8‐2023	19–99	00:33:51
230811_2	11‐8‐2023	18–99	00:36:07
231009	9‐10‐2023	14–94	00:52:24

**TABLE A2:** All species utilized in GBIF downloads, their identifiers (LSID), and number of occurrences in each dataset. Bold and underlined taxa highlight multispecies taxa that were investigated and the total number of species (*n*).

Taxon	LSID	Occurrences in Swedish dataset	Occurrences in global dataset
*Protanthea simplex*	urn:lsid:marinespecies.org:taxname:100913	62	2036
*Sabella pavonina*	urn:lsid:marinespecies.org:taxname:130967	652	910
*Geodia barretti*	urn:lsid:marinespecies.org:taxname:134023	10	361
*Mycale lingua*	urn:lsid:marinespecies.org:taxname:168640	120	309
*Polycarpa pomaria*	urn:lsid:marinespecies.org:taxname:103909	2	26
Serpulidae (*n* = 8)	urn:lsid:marinespecies.org:taxname:988	1913	13,469
*Apomatus globifer*	urn:lsid:marinespecies.org:taxname:238208	9	36
*Chitinopoma serrula*	urn:lsid:marinespecies.org:taxname:130986	8	59
*Filograna implexa*	urn:lsid:marinespecies.org:taxname:130989	431	6270
*Hydroides norvegica*	urn:lsid:marinespecies.org:taxname:131009	241	5631
*Placostegus tridentatus*	urn:lsid:marinespecies.org:taxname:131025	83	413
*Protula tubularia*	urn:lsid:marinespecies.org:taxname:131035	2	28
*Serpula vermicularis*	urn:lsid:marinespecies.org:taxname:131051	112	404
*Spirobranchus triqueter*	urn:lsid:marinespecies.org:taxname:555935	1027	628
*Molgula* spp. (*n* = 2)	urn:lsid:marinespecies.org:taxname:103509	10	21
*M. citrina*	urn:lsid:marinespecies.org:taxname:103775	7	17
*M. complanata*	urn:lsid:marinespecies.org:taxname:103776	3	4
*Ascidia* spp. (*n* = 5)	urn:lsid:marinespecies.org:taxname:103483	316	1163
*A. callosa*	urn:lsid:marinespecies.org:taxname:103700	0	59
*A. mentula*	urn:lsid:marinespecies.org:taxname:103710	120	445
*A. obliqua*	urn:lsid:marinespecies.org:taxname:103713	12	323
*A. prunum*	urn:lsid:marinespecies.org:taxname:103714	0	28
*A. virginea*	urn:lsid:marinespecies.org:taxname:103717	184	308
*Ciona intestinalis*	urn:lsid:marinespecies.org:taxname:103732	594	1353
*Porania pulvillus*	urn:lsid:marinespecies.org:taxname:125166	36	122
*Acesta excavata*	urn:lsid:marinespecies.org:taxname:140232	32	25
Actiniidae (*n* = 3)	urn:lsid:marinespecies.org:taxname:100653	381	1725
*Bolocera tuediae*	urn:lsid:marinespecies.org:taxname:100817	170	1167
*Urticina eques*	urn:lsid:marinespecies.org:taxname:100833	3	123
*Urticina felina*	urn:lsid:marinespecies.org:taxname:100834	208	435
*Munida* spp. (*n* = 2)	urn:lsid:marinespecies.org:taxname:106835	127	807
*M. rugosa*	urn:lsid:marinespecies.org:taxname:107160	42	190
*M. sarsi*	urn:lsid:marinespecies.org:taxname:107163	85	617
*Phakellia ventilabrum*	urn:lsid:marinespecies.org:taxname:132511	99	387
*Lithodes maja*	urn:lsid:marinespecies.org:taxname:107205	25	1319
*Caryophyllia smithii*	urn:lsid:marinespecies.org:taxname:135144	976	4274
*Alcyonium digitatum*	urn:lsid:marinespecies.org:taxname:125333	11,479	13,745
